# FIND integrated biobanks: A network model for efficiency and preparedness based on equitable partnership contracts

**DOI:** 10.1017/cts.2025.10232

**Published:** 2026-01-02

**Authors:** Warren Fransman, Imane El Idrissi, Ruzica Biga, Devy Emperador, Nelson Adedosu, Tatiana Caceres, Johnson Etafo, Elie Ishara-Nshombo, Hugo Kavunga-Membo, Daniel Mukadi-Bamuleka, Milka Muthoni, Ecaterina Noroc, Ntobeko Ntusi, Ephraim Ogbaini-Emovon, Elena Romancenco, Natasha Taylor-Meyer, Carlos Zamudio Fuertes, Abha Saxena, Dominic Allen, Fay Betsou, Emilie Alirol

**Affiliations:** 1 FIND, Geneva, Switzerland; 2 Molecular/Virology Laboratory, Federal Medical Center, Owo, Nigeria; 3 Instituto de Medicina Tropical Alexander von Humboldt, Cayetano Heredia University, Lima, Peru; 4 Rodolphe Mérieux Laboratory, Institut National de Recherche Biomédicale (INRB), Goma, Democratic Republic of the Congo; 5 Sample Management and Biobanking Division, Department of Lab Services, Biosafety and Biobanking, Kenya Medical Research Institute, Nairobi, Kenya; 6 National HIV/STI/VH Reference Laboratory, Infectious Diseases Clinical Hospital “Toma Ciorba”, Chișinău, Republic of Moldova; 7 Division of Cardiology, Department of Medicine, University of Cape Town, Cape Town, South Africa; 8 South African Medical Research Council, Parow Valley, Cape Town, South Africa; 9 Irrua Specialist Teaching Hospital, Irrua, Edo, Nigeria; 10 Division of Biobank, State University of Medicine and Pharmacy “Nicolae Testemitanu”, Chișinău, Republic of Moldova; 11 Independent Bioethics Advisor, Geneva, Switzerland; 12 Independent Biobank Consultant, Dudelange, Luxembourg; 13 Institut Pasteur, CRBIP, Paris, France

**Keywords:** Biobank, network, lmic, partnership, pandemic preparedness

## Abstract

The FIND Integrated Biobank (FIB) network was established to address long-standing barriers in translational research, diagnostic development, and pandemic preparedness, particularly in low- and middle-income countries (LMICs). This article presents the FIB network as an innovative, contract-based model for equitable biobanking partnerships that support rapid, quality-assured access to clinical biospecimens for translational research and diagnostic evaluations. The FIB model offers a replicable and scalable approach to global diagnostic research and preparedness, anchored in equitable, legally binding partnerships. It advances the goals of the WHO Pandemic Agreement and provides a practical pathway for integrating LMIC institutions into global translational research ecosystems.

## Introduction

FIND is an international non-profit organization dedicated to transforming diagnostics and testing to solve some of the world’s most challenging health issues. In recent years, FIND has catalyzed the development of more than 20 innovative diagnostic tools. During the COVID-19 crisis, FIND was a co-convener of the Access to COVID-19 Tools (ACT) Accelerator Diagnostics Partnership, accelerating the development and production of new diagnostic tools, as well as ensuring equitable access to testing, as part of the global response to the pandemic.

Even before COVID-19 hit, the uptake of diagnostic tests in low- and middle-income countries (LMICs) was hampered by numerous constraints, including access to well-characterized clinical specimens and market failures. A strategic collaboration between FIND and the World Health Organization (WHO) to drive universal access to essential tests was formalized in 2020 and became central to the global COVID-19 response. One critical gap in the global COVID-19 response, as is the case more generally in the development of diagnostic tools, was the efficient procurement of fit-for-purpose biospecimens for the evaluation of diagnostic assays. Addressing this gap is critical to ensure preparedness for future pandemics [1] and indeed for the evaluation of new diagnostic assays in general. It has been proposed that a coordinated network of quality-assured clinical laboratories would take pressure off countries facing an outbreak and could provide quick access to clinical samples and data [2].

Existing biospecimen collections are sometimes fit-for-purpose, and a Virtual Biobank Directory (VBD) has been developed to facilitate access to these collections in the area of infectious diseases [3]. Over the years, as part of its diagnostics development activities, FIND has supported approximately 100 diagnostic study sites around the world, mostly in LMICs. Many of these sites performed clinical or laboratory evaluations and often stored collections of specimens for future use in diagnostic performance evaluations. However, these sites would not qualify as biobanks in the sense of the ISO definition, i.e. “legal entities or part of legal entities that perform acquisitioning and storing, together with some or all of the activities related to collection, preparation, preservation, testing, analyzing and distributing defined biological material as well as related information and data” [4]. In 2020, in response to the COVID pandemic, FIND launched a novel concept of a decentralized biobank network, with the objective of establishing “just-in-time” collections of well-characterized disease-specific biological resources. The primary use envisaged for this network was to accelerate timelines for setting up new studies to evaluate and validate diagnostic tools, regardless of whether these studies were performed by industrial or academic users. Here, we present our experiences and lessons learned during the development of the FIND Integrated Biobank network (FIB network).

## FIB network

The concept of the FIB network was born from a desire to create an international network of clinical biobank partners with capacity to initiate specimen collection and biobanking with short lead-times, while ensuring appropriate quality to support robust diagnostic evaluations. The implementation was partly funded by WHO and Unitaid.

### The principle

The core objective of the FIB network is to build regional biobank capacity, ensuring wide geographic coverage so that in the event of an outbreak, there is likely to be a site established in the region affected. Beyond pandemic and epidemic preparedness, the principle is that biobank partners are equipped and their staff trained to further engage with their parent organizations to become institutional biobanks and serve clinical and other biomedical research nationally and regionally.

The biobanks included in the network all have a similar governance framework, use common template protocols, standard operating procedures (SOPs), software, and equipment, and comply with the same quality standards and best practices. This standardization ensures quality all along the specimen value chain, accelerates access to biospecimens, and facilitates comparison of performance evaluation results across different geographies. Access to endemic country sites, confidence in the quality of biospecimens, adequate clinical characterization, and timely fulfillment of sample requests are among the most desirable attributes of biobanks as per a recently conducted survey [5].

### The partnership model

**Figure 1.**
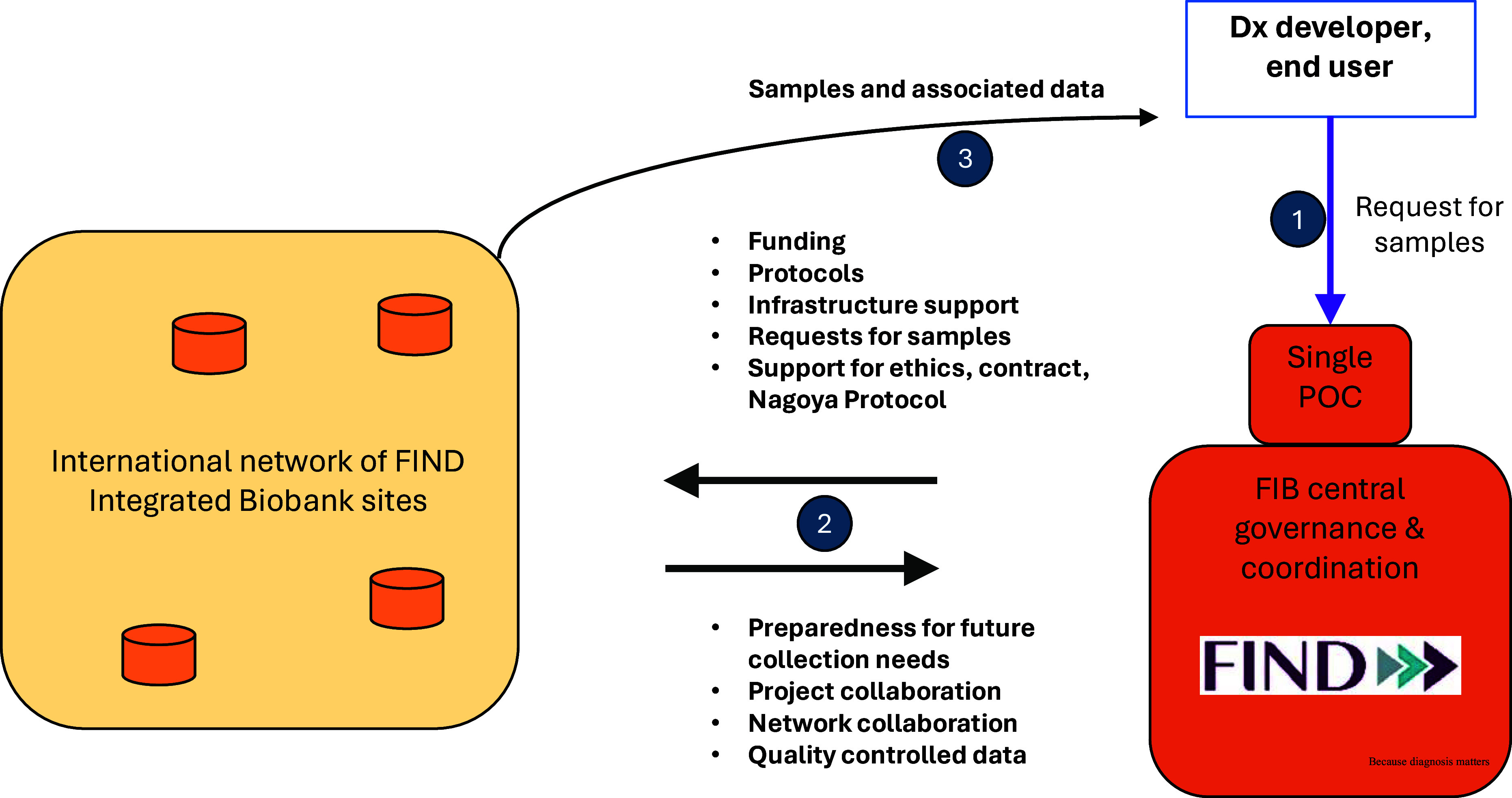
A schematic diagram of the FIB network concept. Numbers in blue circles correspond to the chronological order of operations. FIND = foundation for innovative new diagnostics; FIB = FIND integrated biobank; Dx = diagnostics; POC = point of contact.

The partnership itself is governed by a non-exclusive framework or umbrella agreement between FIND and the biobank site. This encompasses the two critical components of setting up and operating a biobank, i.e. the constitution and handling of collections and the sharing of specimens (Figure 1).

Activities falling under the constitution and handling of collections include the pre-selection of collection sites, the drafting of standardized template documents (e.g. collection forms, informed consent documents), the submissions of those to ethics committees for qualified preapproval of future collections, the development of standard policies and SOPs to ensure consistency and comparability across the sites (Table 1), the definition of standard datasets, the provision of preferred infrastructure items and consumables, and the procurement of a standardized IT solution for specimen and data tracking (OpenSpecimen). FIND prepared an investment case and provides one-off funding to support the site and cover costs of the prospective collection, including costs of equipment required to process and preserve the biospecimens. The range of this initial funding was 200–250k€. FIND provided much of the initial documentation and initial investment and delivered training on best practices as a form of capacity building. Training was both self-paced and face-to-face during live online sessions. Through its public website, FIND also provided an interface with researchers needing samples, facilitating the exchange of information and samples and supporting the contractual aspects of sample distribution. On the other hand, the biobank partner was responsible for local ethics approval and for setting up its operations in line with the agreed SOPs and quality standards. The financial responsibility for the equipment maintenance costs had to be taken on by each site.

**Table 1.** List of FIB documents

Policy and governance documents and associated records:
FIND Policy on access to biological resources and FIND Integrated Biobanks
Registry of contact points for Virtual Biobank Directory and FIB sites
FIND Policy on data protection
Data Protection registry
FIB staff responsibilities
FIB site specificities
FIND best practice on interpretation of Nagoya Protocol
Sample access procedure
Sample request form
Sample request assessment form
FIND MTA template
Contractual documents:
Template framework agreement for FIB
Biobank operational documents and associated records:
Sample plan templates
Collection framework template for IRB
Collection protocol template for IRB
FIB Informed consent template
FIB donor information sheet template
Clinical and biological CRF template
Environmental monitoring and alarm duties
Use and maintenance of freezers
Use and maintenance of liquid nitrogen tanks
Sample identification and pseudonymization
Biobank directory and FIB IT tools
Assessment checklist for Virtual Biobank Directory
Assessment checklist for FIB
FIB procedure for data collection, entry, transfer
General requirements for blood collection
Blood QC CRF
EDTA Blood collection and processing
SST Blood collection and processing
CPT Blood collection and processing
Registry of consumables-PBMC processing
PAXgene RNA blood collection and processing
ccfDNA blood collection and processing
Saliva collection and processing
Saliva QC CRF
Swab collection and processing
Swab QC CRF
Stool collection and processing
Stool QC CRF
Instructions for stool collection
Stool CRF
Urine collection and processing
Urine QC CRF
Storage of samples
Picking and shipment of samples
Quality control documents and associated records:
FIB and biobank directory data management and QC
Biospecimen quality control planning
Biospecimen quality control results

The quality assurance for samples and associated data is based on (i) a procedure for data management and quality control, (ii) quality control CRF forms for each specific type of specimen, (iii) a procedure for biospecimen quality control planning (Table 1). The latter includes both systematic quality control done by the FIB sites (eg., hemolytic index, cell viability) and periodic quality control done by an independent central laboratory (eg., specific cytokine levels). The whole framework and quality plan are based on ISO2387 [4].

The modalities for specimen sharing include the establishment of a specimen access committee, the development of standard contracts and material transfer agreements (MTAs) for end users, support for access and benefit sharing (ABS) documentation if required for prior informed consent (PIC) and mutually agreed terms (MATs), and organization of shipments of specimens to evaluation sites. FIND and the FIB network partner both have votes on the specimen access committee, although neither has a majority or a veto. FIND is responsible for following up on the effective implementation of MATs, e.g. the acknowledgment of parties and co-authorship in publications. When FIND, acting as the single Point Of Contact (POC), organizes sample distribution to third parties, FIND pays for the shipment costs, while no other handling fees are applied to FIB sample distribution. Other features include the option for partners to propose the use of specimens for their own work, encouraging diagnostic manufacturers to use the specimens, requiring specimens to be used in line with FIND’s mission, and the institution of simple cost-recovery fees for the sites, differentiating industrial from academic use.

Specific detailed contracts are signed under the framework or umbrella agreement for each new specimen collection project, for each new evaluation project, and for each new capacity-building project. FIND usually covers the costs of new collections. Scientific publications are under joint authorship.

### Lessons learned and way forward

The FIB network was initially envisaged as a long-term partnership. Continuous support has been provided by FIND to partners since 2020 through the distribution of revised SOPs, templates, and other standard documents; updates to the database and biospecimen management system (OpenSpecimen); support for sample shipments; and delivery of ongoing training.

Current FIB network sites are shown in Table 2. As shown in Table 2, the network includes DRC, Ghana, India, Moldova, Nigeria, Peru, and South Africa. Building the network took three months for the first site in Peru and a further year for the remainder of the sites. During the COVID-19 pandemic, several collection projects have been successfully implemented, enabling the specimen collections described in Table 3.

**Table 2.** Current sites that are partners in the FIB network

Country	Organization
Democratic Republic of the Congo (DRC)	Institut National de Recherche Biomedicale
Ghana	Noguchi Memorial Institute for Medical Research
India	LepraSociety
Moldova	Infectious Diseases Clinical Hospital “Toma Ciorba,” (IDCH)
Nigeria	Federal Medical Centre Owo (FMCO)
Nigeria	Irrua Specialist Teaching Hospital (ISTH)
Peru	Universidad Peruana Cayetano Heradia (UPCH)
South Africa	University of Cape Town (UCT)

**Table 3.** Details of FIB network collections

Site name, country	Number of aliquots	Number of patients/donors	Types of specimens
DCDH, Moldova	25,427	302	Nasal swab, oropharyngeal swab, serum, plasma, saliva
FMCO, Nigeria	3413	196	Whole blood, plasma, serum
INRB, DRC	1756	117	Urine, plasma, serum, oropharyngeal swab, lesion swab
UCT, South Africa	32,667	341	Nasal swab, serum, plasma, whole blood, dried blood spot, saliva, nasopharyngeal swab, dry swab
UPCH, Peru	42,242	502	Nasal swab, oropharyngeal swab, serum, plasma, whole blood, peripheral blood mononuclear cells (PBMC), saliva

As the network has been in existence for 5 years, a few lessons can now be drawn. A key success is that the FIB network overcomes two of the main hurdles encountered when setting up diagnostic development studies during outbreaks: (i) time delays around the initiation and logistics of prospective specimen collections and (ii) governance questions around the use and distribution of biological resources. As there is already an operational framework in place, the network can be ready to start operations on short notice and ensure rapid access to well-characterized specimens. This is possible based on a pre-agreed contractual relationship, pre-arranged ethics approval, pre-defined SOPs, pre-defined consumables, pre-trained staff, and pre-established database, IT system, and data transfer procedures. In effect, biobanks in the FIB network can be operational on short notice in case of outbreaks, conditional only on available funding. Funding is only necessary to cover the direct costs of any new specimen collection needed; the infrastructure and the system are ready.

In addition, an important strength of the FIB framework is that it has proactively addressed ethical challenges that may arise in biobanking in LMICs in the context of pandemics and public health emergencies [6]. This is because significant effort has been devoted to (1) developing suitable informed consent templates, (2) evaluating risk–benefit balance in collection projects, in consultation with local ethics committees, and (3) empowering local sites concerning the use of the biological resources as part of the specimen access procedure.

Global Health R&D is undergoing a paradigm shift whereby core capabilities are being regionalized from drug discovery to clinical trials and vaccine manufacturing. This shift is welcome, and the FIB network is a central element of FIND’s strategic goal of increasing access to diagnostics in LMICs. Most of the FIB network members are also part of larger legal entities, usually clinical sites or laboratories, which also have analytical capabilities. Hence, except if centralized evaluation in a reference laboratory is necessary, FIB network members can perform evaluations of new diagnostics. This possibility is foreseen in the FIB framework agreement and is included in the initial invitation letter.

Finally, one of the greatest strengths of the FIB model is that it is based on contracts, not declarations of willingness. The contracts are entered into freely by mature organizations that have other opportunities for collaborations. Axiomatically, the contracts represent what the parties each consider to be a fair and balanced exchange.

In 2024, an FIB workshop was convened in Nairobi, Kenya, to discuss lessons learned and opportunities for further improvement. All agreed on the need to strengthen the partnership and, at the same time, ensure the network members become further integrated in their countries’ research ecosystems. During the workshop, partners discussed the sustainability and long-term viability of the biobanks and the challenges of cost recovery. Integration of the biobank as an institutional infrastructure, providing services to the hosting institute, was deemed the best way forward. Opportunities were discussed to elevate the partnership from a transactional level (governance, resources and expertise, transparency and accountability, data evidence, and respect) to a collaborative level (shared vision, relationship building, understanding, and trust) [7]. Sites’ principal investigators confirmed the need for strengthening the links between the FIB sites, allowing the sharing of lessons learned among different biobanks. During this workshop, the importance of community outreach activities [6] was also highlighted, and the various sites shared their experiences. Finally, shared values and goals were considered to be key elements in strengthening the network, allowing for the creation of a strong LMIC biobank community of practice.

## Conclusion

In 2020, in response to the COVID-19 pandemic, FIND established a decentralized network of biobanks, the FIND Integrated Biobank (FIB) network. Other publications have reported results of surveys and interviews [8] on the theoretical concept of sustainable biobank networks based on “openness and willingness to share” [9]. The WHO Biohub idea, based on the WHO BioHub System’s Guiding Principles and Terms of Reference also promotes a similar concept, although after 4 years, only 4 countries have signed MTAs, and only 44 viral strains and no human clinical specimens have been shipped so far [10]. The FIB network concept is more than just a theoretical concept. It has been implemented across 6 sites on 4 continents and has delivered concrete results, with 100,000 samples collected, 1100 samples distributed, and 10 diagnostic development projects supported (7 academic, 3 industrial), each based on signed contractual framework agreements and operational contracts. Independently from the COVID-19 collection projects, a collection project on Lassa fever and a collection project on mpox have been supported and have produced around 3000 samples each.

We advocate for this model to be expanded and replicated for efficiency and preparedness whenever prospective specimen collections are needed. Finally, the FIB initiative aligns with the vision set out by the WHO Global Clinical Trials Forum [11] on establishing sustainable research infrastructure in LMICs to support continuous improvement of clinical care and disease prevention, and it also falls squarely within the scope of Article 9 of the recent WHO Pandemic Agreement [12].

## References

[1] Reed T , Waites W , Manheim D , et al. Five Ways that COVID-19 Diagnostics can Save Lives: Prioritizing Uses of Tests to Maximize Cost-Effectiveness. Malaysia: World Bank Group, 2021.

[2] Goncalves A , Peeling RW , Chu MC , et al. Innovative and new approaches to laboratory diagnosis of Zika and dengue: a meeting report. J Infect Dis. 2018;217:1060–1068. 29294035 10.1093/infdis/jix678 PMC6279137

[3] Ongarello S , Fernández Suárez M , Betsou F. The DxConnect Virtual biobank connects diagnostic researchers to clinical samples. Nat Biotechnol. 2022;40:18–19. doi: 10.1038/s41587-021-01168-z. 34937882

[4] International Organization for Standardization. ISO 20387:2018; Biotechnology — Biobanking — General requirements for biobanking, 2018. https://www.iso.org/standard/67888.html, Accessed January 15, 2026.

[5] Batheja D , Goel S , Fransman W , Mantsoki A , Ongarello S , Laxminarayan R. Understanding the value of biobank attributes to researchers using a conjoint experiment. Sci Rep. 2023;13:22728. doi: 10.1038/s41598-023-49394-6. 38123601 PMC10733358

[6] Singh S , Cadigan RJ , Moodley K. Challenges to biobanking in LMICs during COVID-19: time to reconceptualise research ethics guidance for pandemics and public health emergencies? J Med Ethics. 2022;48:466–471. doi: 10.1136/medethics-2020-106858. 33980656 PMC8117471

[7] Schriger SH , Binagwaho A , Keetile M , et al. Hierarchy of qualities in global health partnerships: a path towards equity and sustainability. *BMJ Glob Health*. 2021;6. doi:10.1136/bmjgh-2021-007132. PMC8718486 34969686

[8] Giri J , Pezzi L , Cachay R , et al. Specimen sharing for epidemic preparedness: building a virtual biorepository system from local governance to global partnerships. PLOS Glob Public Health. 2023;3:e0001568. doi: 10.1371/journal.pgph.0001568. 37819913 PMC10566708

[9] Peeling RW , Boeras D , Wilder-Smith A , et al. Need for sustainable biobanking networks for COVID-19 and other diseases of epidemic potential. Lancet Infect Dis. 2020;20:e268–e273. doi: 10.1016/s1473-3099(20)30461-8. 32717208 PMC7380944

[10] World Health Organization. Report on the Pilot Testing Phase on the WHO BioHub System. Geneva: World Health Organization; 2024.

[11] Moorthy V , Abubakar I , Qadri F , et al. The future of the global clinical trial ecosystem: a vision from the first WHO Global Clinical Trials Forum. Lancet. 2024;403:124–126. doi: 10.1016/S0140-6736(23)02798-8. 38128557

[12] 78th World Health Assembly. WHO Pandemic Agreement. https://apps.who.int/gb/ebwha/pdf_files/WHA78/A78_R1-en.pdf, Accessed May 29, 2025.

